# A Cross-Sectional Examination of Patterns of Sedentary Behavior and Cardiometabolic Risk in Community-Dwelling Adults Aged 55 Years and Older

**DOI:** 10.1155/2020/3859472

**Published:** 2020-06-01

**Authors:** Kelsey L. McAlister, Daniela A. Rubin, Koren L. Fisher

**Affiliations:** ^1^Center for Successful Aging, College of Health & Human Development, California State University, Fullerton, CA, USA; ^2^Department of Preventive Medicine, Keck School of Medicine, University of Southern California, Los Angeles, CA, USA; ^3^Department of Kinesiology, College of Health & Human Development, California State University, Fullerton, CA, USA

## Abstract

**Introduction:**

Sedentary behavior (SB) is highly prevalent among older adults, with more than 25% engaging in 6 hours or more of SB daily. SB has been associated with several cardiometabolic biomarkers in younger adults; however, there is a paucity of research in older populations. This study examined associations between patterns of SB and cardiometabolic biomarkers in community-dwelling adults aged 55 years and older.

**Methods:**

Data were drawn from a convenience sample of 54 community-dwelling individuals (12 males, 42 females; mean age = 72.6 ± 6.8 years, range = 56–89 years). Cardiometabolic biomarkers assessed included systolic (SBP) and diastolic blood pressure (DBP), body mass index, waist circumference, and fasting blood glucose and cholesterol parameters. SB was assessed via accelerometry over a 7-day period, and measures included daily time in SB, number and length of sedentary bouts, the number and length of breaks between sedentary bouts, moderate-to-vigorous physical activity (MVPA), and light physical activity (LPA). Associations between the SB measures and each cardiometabolic risk factor were examined using separate stepwise multiple regression models, controlling for sex, MVPA, and accelerometer wear time. Isotemporal substitution models were used to examine the change in cardiometabolic outcomes when SB is replaced by an equal duration of either LPA or MVPA.

**Results:**

Adjusted regression analyses showed that daily sedentary time was positively associated with DBP (*β* = 0.052, *∆R*^2^ = 0.112, *p* = 0.022) and inversely associated with HDL cholesterol (*β* = −0.111, *∆R*^2^ = 0.121, *p* = 0.039). Sedentary bout length was also associated with DBP and HDL cholesterol (*β* = 0.575, *∆R*^2^ = 0.152, *p* = 0.007; *β* = −1.529, *∆R*^2^ = 0.196, *p* = 0.007, respectively). Replacement of 10 minutes of SB a day with LPA was associated with improved DBP and HDL cholesterol (*p* ≤ 0.05). No other significant associations (*p* ≤ 0.05) were found.

**Conclusion:**

Sitting for prolonged periods of time without interruption is unfavorably associated with DBP and HDL cholesterol. Prospective studies should identify causal relationships and observe specific changes in cardiometabolic profiles in older populations.

## 1. Introduction

Physical activity (PA) is understood to be an important factor in healthy aging. Current PA guidelines recommend 150 to 300 minutes of moderate-to-vigorous PA (MVPA) per week along with muscle strengthening activities on at least two days per week [1]. As people age, maintaining sufficient PA levels is especially important as physiological decline begins to accelerate after the age of fifty [2]. Sarcopenic changes in the muscle are associated with a decline in resting metabolic rate and glucose metabolism, contributing to increased fat accumulation and insulin resistance [3, 4]. Over time, these changes may negatively affect blood pressure, metabolic function, and overall cardiovascular health [3, 4]. Physical activity has been shown to attenuate the rate and degree to which these cardiometabolic changes occur [2, 5]. However, despite the well-known health benefits of PA, fewer than 30% of adults over the age of 50 engage in the recommended amount of MVPA [6, 7]. People aged 50 years and older are expected to comprise approximately 40% of the US population in the next 10 years, with the majority managing multiple chronic health conditions [8, 9]. Therefore, the high prevalence of sedentary behavior (SB) among older adults is of significant concern as it likely contributes to the minimization of time spent in PA. Many cardiometabolic outcomes could be improved simply if older adults reduced their SB by increasing the time they spend in light PA (LPA). For many older adults, this is likely a more achievable and realistic goal than increasing time spent in MVPA [10].

While PA is an important strategy for mitigating age-related cardiometabolic changes, recent research suggests that reducing SB among older adults may have important benefits for cardiometabolic health and physical function, independent of PA [7, 11, 12]. Sedentary behavior [those activities performed while seated or lying down during waking hours, where energy expenditure is less than 1.5 metabolic equivalents (METS)] is highly prevalent, particularly among older adults, with evidence suggesting that more than 25% of older people sit for at least 6 hours per day [13–15]. Much of the published research in the older population is based upon self-reported PA and SB data, [12, 16] which may result in the overestimation of PA and underestimation of SB as a result of recall and social desirability biases.

Emerging evidence has shifted toward using objective measures to identify associations between SB and chronic illnesses in older adults [17, 18], but findings regarding cardiometabolic outcomes are inconsistent. A number of studies in older adults have found significant associations between SB and several cardiometabolic markers, including body mass index (BMI), waist circumference (WC), and cholesterol and insulin resistance parameters, independent of PA [14, 19]. On the other hand, Figueiro et al. only found significant associations between SB, systolic blood pressure, and high-density lipoprotein (HDL) and found null associations between SB, WC, glucose, low-density lipoprotein (LDL), and triglycerides in older adults [17]. Other evidence showed that remaining sedentary for most of the day, even while meeting PA guidelines, was associated with negative cardiometabolic effects including glucose regulation and cholesterol parameters [13]. Belletiere et al. found that older women spending the most time in SB were twice as likely to be diagnosed with diabetes, while del Pozo-Cruz et al. showed that replacing SB with LPA or MVPA is associated with reduced WC, fasting insulin, and increased HDL concentration in older individuals [20, 21]. However, Belletiere and colleagues were not able to establish significant associations between breaks in SB and diabetes prevalence [20]. A recent systematic review by Wirth and colleagues (2017) suggested that associations between SB, anthropometrics, and biomarkers are inconclusive in this population due to the mix of significant and insignificant findings in a number of high-quality studies [22]. Although evidence is growing, the influence of SB metrics on cardiometabolic outcomes is still uncertain.

A few studies have evaluated the possible cardiometabolic benefits of replacing SB with PA in older adults [23]. Isotemporal substitution modeling is a relatively new approach to examining behaviors such as PA and SB. A growing body of literature utilizing isotemporal substitution modeling in adult populations suggests a number of benefits of replacing SB with PA [23–25]. Replacing SB with an equivalent amount of LPA and MVPA was associated with lower odds of metabolic syndrome, decreased clustered cardiometabolic risk, and healthier individual cardiometabolic markers in samples of adults ages 29–82 and 50–64 years [24, 25]. In their isotemporal substitution analysis, Ryan et al. showed that replacing one hour per day of MVPA with an equivalent amount of SB is associated with higher triglyceride levels in older adults [26]. However, relatively few studies have used isotemporal substitution modeling in the older adult populations [23]. Statistically modeling the impact of replacing SB with LPA or MVPA in older adults could provide novel insights toward the creation of evidence-based recommendations for appropriate, realistic reallocation of energy balance behaviors in older adults related to cardiometabolic outcomes [23].

Given that older adults are the most sedentary segment of the population, SB may be particularly salient to their cardiovascular and metabolic health. While the body of SB research on older populations is growing, a clear understanding of the relationships between SB and cardiometabolic risk factors in both inactive and active older adults is needed. Understanding the role of SB may contribute to the development of successful interventions. Therefore, the purpose of this study was to cross-sectionally examine objective patterns of SB (daily sedentary time, sedentary bout length, sedentary break length) as they relate to cardiometabolic health in community-dwelling adults aged 55 years and older. We further aimed to model how cardiometabolic health would change if 10 minutes of SB were reallocated to 10 minutes of LPA or MVPA. We hypothesized that less time spent in SB, more SB bouts, and more SB breaks will be positively associated with cardiometabolic health. We also hypothesized that replacing 10 minutes of SB with either LPA or MVPA would be associated with healthier cardiometabolic outcomes.

## 2. Methods

Approval for this study was obtained from the Institutional Review Board at California State University, Fullerton, and informed consent was obtained from all eligible study participants.

### 2.1. Participants

For this observational study, a convenience sample of healthy, community-dwelling adults aged 55 years and older were recruited from two university-affiliated community organizations. Study volunteers were invited to participate if they were living independently, had normal cognitive function, and were able to walk without the use of an assistive device. Individuals with known cognitive disorders and those unable to walk independently were excluded from the study, as were those taking medications (e.g., statins, angiotensin-converting enzyme inhibitors, angiotensin II receptor blockers, diuretics, beta blockers) that could potentially alter metabolic parameters.

### 2.2. Procedures

Following the initial telephone contact, eligible participants were scheduled for an on-campus appointment. Participants were instructed to fast for at least 12 hours prior and to avoid exercise the morning of their appointment. Upon arrival, a research assistant explained the study procedures and obtained informed consent. Participants then rested for a minimum of five minutes prior to the commencement of the physical testing.

### 2.3. Physical Measures

#### 2.3.1. Resting Blood Pressure and Heart Rate

Resting systolic and diastolic blood pressure (SBP and DBP, respectively) were measured using an automated blood pressure monitor (Omron-HEM 705, Heart Rate Monitors, USA). Three measurements, each separated by one minute, were taken in the left arm with participants seated, feet flat on the floor, and back supported. The averages of the three measurements for SBP and DBP were used in the analyses.

#### 2.3.2. Fasting Cholesterol and Glucose Parameters

Finger-prick blood sampling was used to measure fasting cholesterol and glucose parameters using the World Health Organization protocol [27]. Participants were tested using their nondominant hand following a verbally verified fasting period of at least 8 hours. The sample was analyzed immediately for total cholesterol (TC), HDL, non-HDL, and glucose (GLU) concentrations using a Cholestech analyzer (Alere Cholestech LDX Analyzer, San Diego, CA, USA).

#### 2.3.3. Anthropometric Measures

Participants' height was measured to the nearest 0.5 cm and body weight measured to the nearest 0.1 kg using a wall-mounted stadiometer and a digital scale (Ohaus ES200L, Pinewood, NJ, USA), respectively. These values were then used to calculate BMI. Waist circumference (WC) was measured to the nearest 0.5 cm, following the World Health Organization standard procedures [28]. Two measurements were recorded, and the average WC was used in the analyses.

#### 2.3.4. Physical Activity and Sedentary Behavior

Physical activity and SB were objectively measured using a waist-worn triaxial accelerometer (Actigraph GT3X, Pensacola, FL, USA). Participants were instructed to wear the accelerometer on the right hip during waking hours for seven consecutive days, removing it only for showering/bathing, swimming, and sleeping. A written log was provided so that participants could record contextual information describing the time of day and reasons for device removal.

The accelerometer recorded data on temporal patterns of PA and SB, including duration, frequency, and intensity of activity in 5 second epochs. Wear time compliance was defined as a minimum of 8 hours per day on a minimum of 4 days, including at least 1 weekend day. Nonwear time was defined as any period of 60 minutes or longer where no activity was recorded [29]. Cut-points established for adults aged 20 years and older by Troiano et al. were used to define SB (0–99 counts), LPA (100–2019 counts), moderate PA (2020–5998 counts), and vigorous PA (5999 counts and above) [30]. The variables derived from the accelerometry data included minutes per day of LPA, MVPA, and SB, along with the number and average length (in minutes) of daily sedentary bouts and sedentary breaks. A sedentary bout was defined as 10 or more minutes of consecutive accelerometer readings <100 cpm, and a sedentary break was defined as a nonsedentary period between two sedentary bouts.

#### 2.3.5. Statistical Methods

Analyses were completed using the Statistical Package for the Social Sciences (SPSS Version 26). The level of statistical significance was set as *p* < 0.05. For descriptive purposes, participants were classified as “at risk” or “not at risk” for the eight cardiometabolic parameters based on standard American Heart Association criteria [31]. Cut-points for each cardiometabolic marker for the “at risk” category include the following: BMI ≥ 25 kg/m^2^, WC ≥ 88 cm for females or ≥102 cm for males, SBP ≥ 140 and/or DBP ≥ 90 mmHg, TC ≥ 200 mg/dL, HDL ≤ 40 mg/dL, non-HDL ≥ 130 mg/dL, and blood glucose ≥ 100 mg/dL. A 3-level categorical variable indicating the number of cardiometabolic risk factors (none; 1 to 2; 3 or more) and a dichotomous variable identifying those who met the current PA guidelines (minimum of 150 minutes per week of moderate PA or 75 minutes per week of vigorous PA) were also computed. Muscle strengthening guidelines were not included in identifying those who met the current PA guidelines versus those who did not, as the accelerometer could not capture the amount of muscle strengthening activities a participant engaged in.

Participant characteristics were examined using frequencies and proportions or means ± SD, as appropriate. For analytical purposes, certain categorical sociodemographic variables (ethnicity, education, and income) were collapsed. Ethnicity was collapsed into a three-level variable (White, non-White, missing). Education was recoded from a 9-level variable to a 3-level variable [high school diploma/some college (no degree); Associate's/Bachelor's degree; professional/graduate degree (professional degree, Master's, and/or Doctorate)]. The 12-level income variable was collapsed into three levels (<$30,000; $30,000–$89,999; and ≥$90,000). Following data reduction, the continuous variables were screened for normality, and no violations of this assumption were evident.

Bivariate Pearson correlations were used to determine the associations between each cardiometabolic and continuous PA variable and among the continuous PA variables. Associations between daily time in SB and the number and length of sedentary bouts and breaks, and each cardiometabolic health parameter were examined using separate stepwise multiple regression models. All models were adjusted for the known confounders of age, sex, MVPA, and accelerometer wear time if appropriate. These covariates were added at the first step with the SB variables and then added to the model at the second step.

To examine the potential impact of replacing 10 minutes of SB with 10 minutes of LPA and MVPA, linear regression modeling with isotemporal substitution was used [32]. All activity variables (LPA and MVPA) were entered into the models simultaneously, along with total wear time (i.e., SB + LPA + MVPA). Sedentary behavior, as the variable of interest, was excluded from the model. Prior to entry into the models, LPA and MVPA were each divided by a constant of 10 such that a unit increase represented an increase of 10 minutes a day within the given variable. Total wear time is constrained to waking hours; therefore, the resulting regression coefficients represent the effect of reallocating a 10 minutes bout of SB to an equal time bout of LPA or MVPA, without consideration of sleep. All isotemporal substitution models were adjusted for age.

## 3. Results

### 3.1. Participant Characteristics

Fifty-four community-dwelling older adults (12 males, 42 females; mean age = 72.7 ± 6.7 years, range 56–89 years) participated in this study. Valid accelerometer data and complete blood data were obtained for 49 and 41 out of 54 participants, respectively.

The participant characteristics are summarized in Table 1. The majority of participants were White (78%), had completed at least one postsecondary degree (74%), and reported an annual household income of more than $90,000 per year (39%). The majority of participants rated their health as “very good” or “excellent” (70%) while at least 50% had elevated blood pressure (55.6%) and/or elevated WC (50%). The mean BMI and WC were 25.9 ± 4.5 kg/m^2^ and 90.4 ± 13.9 cm, respectively, while SBP and DBP averaged 134.1 ± 18.2 mmHg and 74.9 ± 9.0 mmHg, respectively. The mean HDL and TC concentrations were 52.5 ± 17.0 mg/dL and 174.7 ± 35.7 mg/dL, respectively, and fasting GLU averaged 95.8 ± 14.3 mg/dL. Even without complete blood data for all participants, close to 30% of participants were classified as having 3 or more cardiometabolic risk factors, and more than 25% met the diagnostic criteria for metabolic syndrome. Lastly, participants accrued approximately 274 minutes of LPA and 23 minutes of MVPA daily and spent approximately 10 hours per day engaged in SB. The average length of a sedentary bout was 26 minutes, with the average break in SB lasting 65 minutes.

Systolic blood pressure was significantly correlated with SB (*r* = 0.391, *p* < 0.01), and non-HDL cholesterol was significantly correlated with LPA (*r* = −0.335; *p* < 0.05). The remaining correlations between the cardiometabolic and PA variables were not statistically significant and were weak to moderate in strength (*r* < 0.59). Among the PA variables, SB was significantly correlated with both LPA (*r* = −0.331, *p* < 0.05) and MVPA (*r* = −0.520, *p* < 0.001). There were no other significant correlations among the PA variables, and given that all correlations were *r* < 0.6, multicollinearity between the PA variables is unlikely to be an issue in the regression analyses.

### 3.2. Regression Analyses

Preliminary analyses revealed that there were no differences between men and women on any SB or PA measures, so sex was not included as a control variable in the regression analyses. After adjusting for covariates, daily time in SB was positively associated with DBP (*β* = 0.052, *∆R*^2^ = 0.112, *p* = 0.022) and inversely associated with HDL cholesterol (*β* = −0.111, *∆R*^2^ = 0.121, *p* = 0.039; see Table 2). Sedentary bout length was also significantly associated with DBP (*β* = 0.575, *∆R*^2^ = 0.152, *p* = 0.007) and HDL cholesterol (*β* = −1.529, *∆R*^2^ = 0.196, *p* = 0.007; see Table 2). No other significant associations were found between daily time in SB, sedentary bout length, sedentary break length, and any cardiometabolic health parameter (p > 0.05 for all); however (*p* = 0.05 for all), associations between daily SB and SBP (*p* = 0.072) and those between sedentary bout length and both BMI and WC (*p* = 0.064 and *p* = 0.070, respectively) did approach statistical significance.

**Figure 1:**
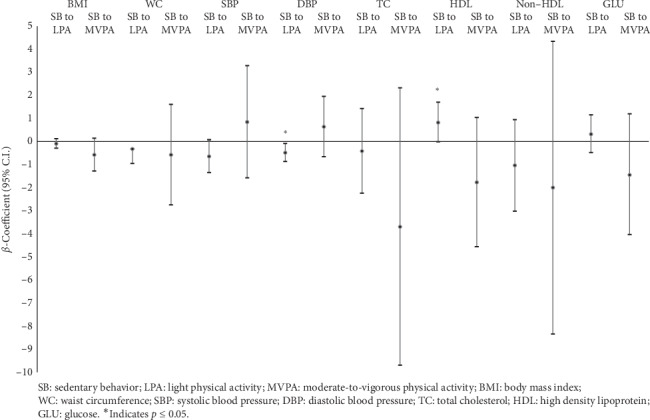
Isotemporal substitution models for cardiometabolic outcomes in response to 10 minutes per day substitution of SB with LPA or MVPA. Values shown are *β* (95% CI) and are adjusted for age

The *β*-coefficients obtained from the isotemporal substitution analysis are presented in Table 3. Replacing just 10 minutes a day of SB with an equivalent amount of LPA was associated with a 0.09% decrease in DBP (*β* = 0.091; *p* < 0.05) and a 0.84% increase in HDL (*β* = 0.843; *p* = 0.05). The results of isotemporal substitution modeling produced associations in the expected direction for WC (*β* = −0.32), SBP (*β* = −0.642), TC (*β* = −0.405), and non-HDL cholesterol (*β* = −1.035) when SB was replaced with LPA, and for BMI (*β* = −0.573), WC (*β* = −0.565), TC (*β* = −3.690), non-HDL (*β* = −1.995), and GLU (*β* = −1.436) when SB was replaced with MVPA; however, these associations were not statistically significant (*p* < 0.05 for all). To better visualize the results, the same models are also presented in Figure 1.

## 4. Discussion

This study examined relationships between daily sedentary time, patterns of SB, and several cardiometabolic outcomes in adults aged 55 years and older. With the exception of blood glucose levels, the associations were in the expected direction; however, few were statistically significant. Greater sedentary time, longer sedentary bout length, and shorter break length were associated with higher DBP (but not SBP), even after adjusting for selected confounders. The consistent associations between the measures of SB and DBP are in line with earlier studies showing positive associations between self-reported and objectively measured sedentary time and DBP in adult populations [33, 34]. However, findings from studies examining relationships between SB and DBP in older adults have been null [14, 35]. Furthermore, few studies have considered the pattern of SB (number and length of sedentary bouts and breaks) in relation to blood pressure and other cardiometabolic parameters and, thus, our understanding of these relationships in older adults remains limited [33].

Interestingly, our results suggest that bout length may have a bigger influence on DBP compared to overall time spent in SB. Our estimates suggest that, per every 1-minute increase in total SB, DBP would increase by 0.05 mmHg, whereas for every 1-minute increase in SB bout length, DBP would increase by 0.58 mmHg. We further showed that replacing just 10 minutes of SB with 10 minutes of LPA would result in a significant improvement in DBP. These findings suggest that the length of SB bouts may be a more important contributor to DBP than total SB time during the day. However, SB was not significantly associated with SBP in the present study, as it has been in others, so further research is needed to better understand the relationships between SB and blood pressure in older adults and whether there is a minimum threshold for sedentary bout length that is associated with worsening of blood pressure [34].

In the present study, we showed that greater sedentary time and longer sedentary bout length were also associated with lower levels of HDL cholesterol. In their recent systematic review, Wirth and colleagues likewise showed that high SB was unfavorably associated with HDL [22]. Our results further corroborate findings from Figueiro and colleagues, who found inverse associations between SB and HDL in older adults [17]. Considering that older adults are more likely to increase their levels of LPA than MVPA when reducing SB [36], our findings suggest that LPA has a greater influence over HDL concentrations in older adults than has been previously understood. Ekelund et al. recently showed that greater amounts of PA at any intensity and less sedentary time were associated with a reduced risk of premature mortality in older adults in a dose-response pattern, which provides further support for the potential role of LPA in counteracting negative shifts in HDL levels (and DBP) that are associated with age [12].

Like our results regarding SB and DBP, we found a greater parameter estimate for SB bout length with HDL than we did for overall time spent in SB with HDL. Per every 1-minute increase in overall time spent in SB, we estimated that HDL would decrease by 0.11 mg/dL, whereas per every 1-minute increase in SB bout length, HDL would decrease by 1.53 mg/dL. It is possible that breaking up prolonged bouts of SB may better influence HDL than simply reducing the total amount of time in SB. This is further supported by our results showing that substituting 10 minutes of SB with an equal duration of LPA would result in improved HDL cholesterol levels. Future studies should consider examining the effects of various patterns of SB on HDL, as it appears that the way in which SB is accumulated may have a stronger influence on HDL concentrations compared to overall total SB.

None of the SB measures in the present study were significantly associated with BMI, WC, or any blood parameters, apart from HDL. In studies of older adults, Vallance et al. and Reid et al. showed greater sitting time to be associated with higher BMI and WC, lower levels of lean mass, higher fat mass, and higher plasma glucose in older adults. [37, 38] Gennuso et al. found that SB was significantly associated with BMI, WC, and plasma glucose, suggesting that reducing SB could benefit overall body composition in people aged 55 years and older [14]. Wirth and colleagues cautioned that there is insufficient evidence of an association between SB and blood lipids or glycemic parameters, arguing that heterogeneity in definitions of SB and the diverse range of reported outcomes makes it difficult to develop a clear understanding of these relationships in older adults [22].

Our study adds to the body of literature pertaining to SB and cardiometabolic health outcomes in older adults and is one of a limited number to demonstrate that sitting for prolonged periods of time may influence DBP. The observational nature of our study limits our ability to establish causal links between the SB measures and the cardiometabolic parameters. We were unable to obtain complete accelerometer data from all participants due to lack of wear time compliance as well as accelerometer malfunction. Likewise, we were unable to obtain adequate blood samples from all participants due to dehydration, cold hands, or lack of blood flow to the pricked finger. We did not collect sleep data and so our statistical models do not reflect a 24-hour cycle but consider only activity performed during waking hours. Our relatively small sample size limited our ability to control for all potential confounders in the analyses. Although post hoc power calculations showed that our study achieved 90% power to detect large effect sizes, there was insufficient statistical power to detect small effect sizes which may explain the lack of significant associations in some analyses. Nonetheless, our findings contribute to the evidence base of the relationships between SB and cardiometabolic parameters in older adults.

This study provides novel insights regarding the potential benefit of activity reallocation in an older adult population, which future studies should consider incorporating in a larger sample. There is a considerable need for prospective cohort and randomized controlled studies to better elucidate the causal relationships to determine if reducing SB in older people is effective in improving their cardiometabolic profile and/or level of cardiovascular disease. In the meantime, health practitioners should encourage older patients to reduce both the total amount of time they spend in SB and the length of their sedentary bouts, in addition to recommending that they increase their PA.

## Figures and Tables

**Table 1:** Participant characteristics (*N* = 54).

	Mean ± SD
Age	72.7 ± 6.7
	*n* (%)
Sex	
Male	12 (22.2)
Female	42 (77.8)
Ethnicity	
White	42 (77.8)
Hispanic/Asian	6 (11.1)
Missing	6 (11.1)
Education	
High school diploma/some college	13 (24.1)
Associate's/Bachelor's degree	22 (40.7)
Professional/graduate degree	18 (33.3)
Missing	1 (1.9)

Annual household income	
<$30,000	7 (13.0)
$30,000–$89,999	19 (35.2)
≥$90,000	21 (38.9)
Missing	7 (13.0)
Self-reported health	
Excellent	13 (24.1)
Very good	25 (46.3)
Good	15 (27.8)
Fair/poor	1 (1.9)
Missing	0 (0.0)
Physical activity (*n* = 49)	
Meeting PA guidelines	*n* (%)
Yes	13 (24.1)
No	36 (75.9)
	Mean ± SD
LPA (min/day)	274.4 ± 73.6
MVPA (min/day)	23.3 ± 20.1
Total SB (min/day)	606.2 ± 88.1
Number of sedentary bouts (per day)	16.0 ± 3.0
Sedentary bout length (min)	25.9 ± 5.9
Number of sedentary breaks (per day)	15.8 ± 3.0
Sedentary break length (min)	64.5 ± 25.6

Cardiometabolic parameters	
	Mean ± SD
Height (cm)	164.3 ± 9.1
Weight (kg)	70.1 ± 14.2
BMI (kg/m^2^)	25.9 ± 4.5
WC (cm)	90.4 ± 13.9
Blood pressure	
SBP (mmHg)	134.1 ± 18.2
DBP (mmHg)	74.9 ± 9.0
Blood Parameters (*n* = 42)	
HDL (mg/dL)	52.5 ± 17.0
^b^Non-HDL (mg/dL)	120.8 ± 37.8
TC (mg/dL)	174.7 ± 35.7
GLU (mg/dL)	95.8 ± 14.3
Cardiometabolic risk factors	*n* (%)
High BMI (≥25 kg/m^2^)	13 (24.1)
Large WC (≥88 cm or 102 cm, females/males respectively)	27 (50.0)
Hypertension (SBP ≥ 140 mmHg and/or DBP ≥ 90 mmHg)	30 (55.6)
^a^Elevated TC (≥200 mg/dL)	9 (16.7)
^a^Low HDL (≤40 mg/dL)	8 (14.8)
^b^Elevated non-HDL (≥130 mg/dL)	12 (22.2)
^a^Elevated GLU (≥100 mg/dL)	11 (20.4)
Number of risk factors for CVD (*n* = 41)	
None	8 (14.8)
1 to 2	17 (31.5)
3 or more	16 (29.6)

^a^ *n* = 41; ^b^*n* = 41. Valid accelerometer data were available for 49 of the 54 participants. Valid data for blood parameters were available for 42 of the 54 participants. The number of risk factors for CVD was calculated only for those participants with complete risk factor data. PA: physical activity; LPA: light physical activity; MVPA: moderate-to-vigorous physical activity; SB: sedentary behavior; BMI: body mass index; WC: waist circumference; SBP: systolic blood pressure; DBP: diastolic blood pressure; HDL: high density lipoprotein; TC: total cholesterol; GLU: glucose; CVD: cardiovascular disease.

**Table 2:** Associations between SB measures and selected cardiometabolic biomarkers.

	BMI			WC			SBP			DBP		
	*β* (95% CI)	*R* ^2^_adj_	*∆R* ^2^	*β* (95% CI)	*R* ^2^_adj_	*∆R* ^2^	*β* (95% CI)	*R* ^2^_adj_	*∆R* ^2^	*β* (95% CI)	*R* ^2^_adj_	*∆R* ^2^
Total SB (min/day)	0.010 (−0.014, 0.034)	0.017	0.014	0.044 (−0.029, 0.118)	−0.020	0.031	0.077 (−0.007, 0.162)	0.251	0.053	0.052 (0.008, 0.096)	0.035	0.112^*∗*^
Number of sedentary bouts												
# (bouts/day)	−0.260 (−0.839, 0.319)	0.021	0.017	−0.296 (−2.096, 1.505)	−051	0.002	0.521 (−1.577, 2.619)	0.198	0.004	−0.023 (−1.152, 1.107)	−0.087	0.000
Bout length (min)	0.210 (−0.013, 0.432)	0.155	0.069	0.634 (−0.053, 0.1.322)	0.023	0.070	0.513 (−0.306, 1.332)	0.222	0.026	0.575 (0.164, 0.987)	0.079	0.152^*∗∗*^
Number of sedentary breaks												
(# breaks/day)	−0.260 (−0.839, 0.319)	0.021	0.017	−0.297 (−2.098, 1.503)	−0.051	0.002	0.516 (−1.583, 2.614)	0.198	0.004	−0.022 (−1.151, 1.107)	−0.087	0.002
Break length (min)	0.016 (−0.055, 0.088)	0.007	0.004	0.020 (−0.201, 0.241)	−0.053	0.001	−0.048 (−0.305, 0.210)	0.196	0.002	−0.081 (−0.218, 0.055)	−0.053	0.031

	TC (*n* = 38)			HDL (*n* = 38)			Non-HDL (*n* = 37)			GLU (*n* = 38)		
	*β* (95% CI)	*R* ^2^_adj_	*∆R* ^2^	*β* (95% CI)	*R* ^2^_adj_	*∆R* ^2^	*β* (95% CI)	*R* ^2^_adj_	*∆R* ^2^	*β* (95% CI)	*R* ^2^_adj_	*∆R* ^2^

Sedentary time (min/day)	0.086 (−0.140, 0.311)	0.078	0.015	−0.111 (−0.215, 0.−006)	0.033	0.121^*∗*^	0.173 (−0.067, 0.414)	0.067	0.056	−0.030 (−0.131, 0.070)	−0.079	0.011
Sedentary bouts												
# bouts/day	2.371 (−2.710, 7.451)	0.087	0.022	1.153 (−1.344, 3.651)	−0.074	0.026	1.989 (−3.559, 7.537)	0.020	0.015	−0.234 (−2.527, 2.059)	−0.090	0.001
Bout length (min)	−0.498 (−2.962, 1.966)	0.066	0.004	−1.529 (−2.615, −0.442)	0.117	0.196^*∗∗*^	0.309 (−2.748, 3.366)	0.005	0.001	−0.062 (−1.162, 1.038)	−0.091	0.000
Sedentary breaks												
# breaks/day	2.2373 (−2.709, 7.455)	0.087	0.022	1.158 (−1.340, 3.656)	−0.073	0.026	1.986 (−3.564, 7.535)	0.020	0.014	−0.235 (−2.529, 2.058)	−0.090	0.001
Break length (min)	−0.113 (−0.723, 0.498)	0.066	0.004	−0.0571 (−0.357, 0.243)	−0.097	0.004	−0.062 (−0.712, 0.588)	0.005	0.001	0.012 (−0.261, 0.284)	−0.091	0.000

^*∗*^ *p* < 0.05; ^*∗∗*^*p* < 0.01; all models were adjusted for age, wear time, and MVPA. BMI: body mass index; WC: waist circumference; SBP: systolic blood pressure; DBP: diastolic blood pressure; TC: total cholesterol; HDL: high-density lipoprotein; GLU: glucose; SB: sedentary behavior; MVPA: moderate-to-vigorous physical activity.

**Table 3:** Isotemporal substitution of 10 minutes of daily SB with equal amounts of LPA and MVPA.

Outcome	SB with LPA			SB with MVPA		
	*β* (95% CI)		*p*-value	*β* (95% CI)		*p*-value
BMI (kg/m^2^)	−0.091	(−0.30; 0.12)	0.388	−0.573	(−1.29; 0.14)	0.115
WC (cm)	−0.320	(−0.96; 0.32)	0.325	−0.565	(−2.75; 1.62)	0.612
SBP (mmHg)	−0.642	(−1.35; 0.07)	0.075	0.856	(−1.57; 3.28)	0.489
DBP (mmHg)	−0.472	(−0.85; −0.09)	0.015	0.642	(−0.67; 1.95)	0.337
TC (mg/dL)	−0.405	(−2.24; 1.43)	0.666	−3.690	(−9.70; 2.32)	0.229
HDL (mg/dL)	0.843	(−0.01; 1.70)	0.053	−1.758	(−4.55; 1.04)	0.218
Non-HDL (mg/dL)	−1.035	(−3.02; 0.95)	0.307	−1.995	(−8.33; 4.34)	0.537
GLU (mg/dL)	0.335	(−0.48; 1.15)	0.418	−1.436	(−4.03; 1.21)	0.288

Regression coefficients are age-adjusted. BMI: body mass index; WC: waist circumference; SBP: systolic blood pressure; DBP: diastolic blood pressure; TC: total cholesterol; HDL: high-density lipoprotein; GLU: glucose; SB: sedentary behavior; LPA: light physical activity; MVPA: moderate-to-vigorous physical activity.

## Data Availability

The data used to support the findings in this study are available from the corresponding author upon request.
